# 1575. Screening Plus Outreach Versus Referrals to Identify Patients for Outpatient COVID Medications

**DOI:** 10.1093/ofid/ofac492.104

**Published:** 2022-12-15

**Authors:** Simon L Parzen-Johnson, Shan Sun, Sameer Patel

**Affiliations:** Ann and Robert H. Lurie Children's Hospital, Chicago, IL; Ann and Robert H. Lurie Children's Hospital, Chicago, IL; Ann and Robert H. Lurie Children's Hospital, Chicago, IL

## Abstract

**Background:**

Scarcity of therapeutics to treat Coronavirus Disease 2019 (COVID-19) during the surge in cases caused by the Omicron variant raised concerns that structural inequities would decrease access to these medications for racial minorities and patients with lower socioeconomic status. We hypothesized that screening plus outreach would increase identification of eligible patients in these vulnerable patient populations when compared to referrals alone.

**Methods:**

A retrospective cohort study of COVID medication allocation was performed at a quaternary pediatric medical care facility between 1/1/22–2/15/22. The two cohorts were patients referred for COVID therapy and patients identified via screening followed by outreach. Screening plus outreach included daily review of laboratory reports for new positive cases of SARS-CoV-2, followed by chart review for high-risk conditions, and communication with providers or directly with eligible patients to offer therapy. Demographic characteristics, chronic medical conditions, socioeconomic parameters, and medication receipt were compared between the two groups.

**Results:**

Overall, 51 and 94 patients were identified via referral and screening plus outreach, respectively. Thirty-two patients received medication of which eight (25%) were identified by screening plus outreach alone. Compared to referred patients, patients in the screen plus outreach group were more likely to have moderate, low, or very low childhood opportunity index (COI) scores (74.5% vs 52.9%%, p = 0.009); public health insurance (69.1% vs 37.5%, p < 0.001); asthma/obesity (63.8% vs 21.6%, p < 0.001), and race/ethnicity other than White, Non-Hispanic (79.8% vs 54.9%, p = 0.002). Patients in the referral group were more likely to receive medication (47.1% vs. 8.5%, p < 0.001).

**Conclusion:**

Compared to referral, screening plus outreach for COVID medications can increase identification of patients who are racial minorities, have asthma/obesity, and have lower socioeconomic status. Future studies should investigate communication strategies to improve uptake of these medications after outreach.

**Disclosures:**

**All Authors**: No reported disclosures.

